# A Case of Abortion, the Fœtus Having Been Retained Four Months after Its Death

**Published:** 1846-02

**Authors:** Thomas C. Osborne

**Affiliations:** Erie, Ala.


**Art. II.**—*A Case of Abortion, the Fœtus having been retained four months after its death.* By Dr. Thos. C. Osborne, of Erie, Ala.

Mrs. E. W., a highly respectable lady of this place, aged 30 years, of a corpulent and sanguine habit, is the mother of three living children; she suffered an abortion two years ago caused by a stubborn chronic hepatitis. Her health improved from that time until the first of April, 1845, when she became satisfied that she was again pregnant, the evidences of which continued until the first of August, at which time, as she reckoned, she was four months advanced in utero-gestation. At this time, without any appreciable cause, she was attacked with pains in the back, &c., attended with contractions of the uterus, and a slight hemorrhage, of the character and consistence of the menstrual fluid. This condition, which lasted several days, was checked, promptly, by a few portions of acetate of lead and opium; but from the time of the attack until the embryo was expelled, three months afterwards, she never experienced a single symptom of pregnancy, neither was there, during that time, a return of the catamenia. This, together with an astonishing increase of corpulency, gave rise in her mind to the most painful apprehensions. She complained of strange and distressing sensations.

On the 27th of the following November, she discovered what she again thought to be the menses; but as the day advanced, the fluid assumed more the appearance of a hemorrhage, and, severe labor pains coming on, she was obliged to take to her bed. For ten hours the efforts of the uterus were almost intolerable, hardly allowing a minute's intermission from pain. Her strength was nearly exhausted, and although repeated doses of tincture of opium were administered, and copious venesection resorted to, the pains persisted, with alarming obstinacy, until the ovum was expelled.

This, upon examination, presented the following appearances:—Membranes thick, firm, and entire, containing five or six ounces of very turbid liquor amnii, and an embryo of full four month's development, in a state of incipient putrefaction. The head was large; the eyes, ears, nose, and mouth distinct; the extremities fully formed with the exception of the nails; the genitals very distinct; the entire length of the embryo being about six inches. The funis umbilicalis was 13 to 15 inches long, and arose from one side, instead of the centre, of the placenta. The fœtal surface of the placenta presented nothing further unnatural, but its uterine surface exhibited many abnormal appearances. In diameter it was about three inches; two-thirds of which was occupied by three distinct portions of a cartilaginous or fibro-cartilaginous substance; and the remaining third was taken up by a substance resembling an old clot of blood. Each portion of these masses was separated from the others by a furrow corresponding to the depth of the disease.

The largest, and yellowish portion of cartilaginous substance, was nearly as firm as the cartilage of the ribs. The next to it, was white, but pervaded by a slight tinge of red, and throughout it was the most granular substance I have ever seen. The third was of a dull white color, and felt under the knife like the substance of the mammary gland. The whole diseased surface was about four lines in thickness, and it was under the clotted part that the funis took its origin.

I have but few remarks to make on the above case.

Mrs. W. was certainly pregnant from the first of April to the first of August. She did not then abort, but if she had done so, there was not time, between the first of August and the 27th of November, for another fœtus to have reached such a stage of development as that attained by the embryo in this case. A complete interruption in the nutrition of the ovum must have taken place at the fourth month of gestation, and the disease of the placenta was the growth of the subsequent months.

December 28th, 1845.

